# Real-time technical support for guiding remotely ICD/CRT-D implantation

**DOI:** 10.3389/fcvm.2025.1525151

**Published:** 2025-03-07

**Authors:** Antonio Curcio, Letizia R. Romano, Florinda M. Augusto, Giovanni Canino, Elisa Coluccio, Alberto Polimeni, Ciro Indolfi

**Affiliations:** ^1^ Department of Pharmacy, Health and Nutritional Sciences, University of Calabria, Rende, Italy; ^2^ Division of Cardiology, Annunziata Hospital, Cosenza, Italy; ^3^ Division of Cardiology, Department of Medical and Surgical Sciences, Magna Graecia University, Catanzaro, Italy; ^4^ Department of Electronics, Information and Bioengineering, Politecnico di Milano, Milan, Italy

**Keywords:** electrophysiology, telemedicine, heart failure, device, technical support, remote monitoring

## Abstract

**Background:**

In the electrophysiologic (EP) lab, technical support for implantable cardioverter/defibrillators (ICD) and cardiac resynchronization therapy (CRT-D) procedures is often limited by the availability and costs of field clinical specialist (FCS) bioengineers.

**Methods:**

This study explores the viability of using remote support through an internet-based platform for ICD and CRT-D implantation procedures, aiming to enhance efficiency and overcome geographical or pandemic-related barriers. After preclinical phases, thirty patients underwent ICD/CRT-D guided either remotely or with on-site FCS implantation at two primary cardiac care centers, with ten procedures guided remotely and twenty cases with on-site FCS.

**Results:**

All procedures in both study arms were successfully completed (100% of cases). Procedural time was shorter in the telemedicine group (*P* = 0.031). Although fluoroscopic time was slightly reduced in the remote guided group, the difference did not reach statistical significance (*P* = 0.5). No major adverse events occurred.

**Conclusion:**

The study demonstrates the feasibility of remotely supported ICD and CRT-D implantation procedures.

## 1 Introduction

Cardiac electrophysiology (EP) has recently faced a series of improvements that potentially are transforming this discipline (1). Evolution in electrode designs, development of new ablative techniques that parallel the clinical results of radiofrequency energy, technical amelioration of cardiac pacemakers (PM) and implantable cardioverter-defibrillator (ICD), combination of multiple parameters and sensors to cardiac resynchronization therapy defibrillators (CRT-D) devices represent some of the main contributors to the growth of the EP field (2, 3).

Field clinical specialist (FCS) biomedical engineer plays a crucial role in the success of implant procedures in EP labs, ensuring that all technologies function correctly and patients receive top-notch care. Their expertise minimizes risks and ensures that implanted devices meet clinical needs through the following steps:

• Preparation and configuration of equipment
• Technical support during the procedure
• Assistance in device programming
• Training and support for staff
• Documentation and reporting

Improvements to the software and novel functions of cardiac rhythm devices require specialized knowledge. Continuous training for physicians and hospital staff is costly and difficult to manage. Therefore, having a FCS in the EP lab is generally expected and considered best practice. The specifics can vary by hospital and regional guidelines.

Nonetheless, the most intriguing contributions come from remote monitoring (RM) of previously implanted devices (4), since this technology — acknowledged among the evolving fields of telemedicine in cardiology (5) — allows, in theory, a continuous supervision and follow-up of virtually all the patients who have been discharged from a certain hospital (6). Whereas the steps following patients discharge such as device RM and outpatients visit scheduling are easily performed (7), yet no proofs regarding the first phases of implantation are available (8). Such lack of knowledge refers to the difficult tasks usually encountered during appropriate patients’ selection (9), or adequate materials and supplies choice (10, 11), or demanding procedures like ICD implantation in secondary prevention, coronary sinus selective engagement for CRT-D, or upgrading interventions (12).

Therefore, considerable accessibility barriers remain in the form of on-site need of a FCS biomedical engineer during the EP lab procedures since such technical personnel has access to appropriate technology and is required to be promptly available in all hospitals at any moment (13). For these reasons, we sought to investigate whether ICD/CRT-D could be easily implanted without the presence of the FCS in the EP lab during our routine daily clinical activity.

## 2 Methods

This pilot study (NCT06404021) was conducted at the University of Catanzaro and the University of Calabria, two primary care cardiac centers located in Italy.

**Figure 1:**
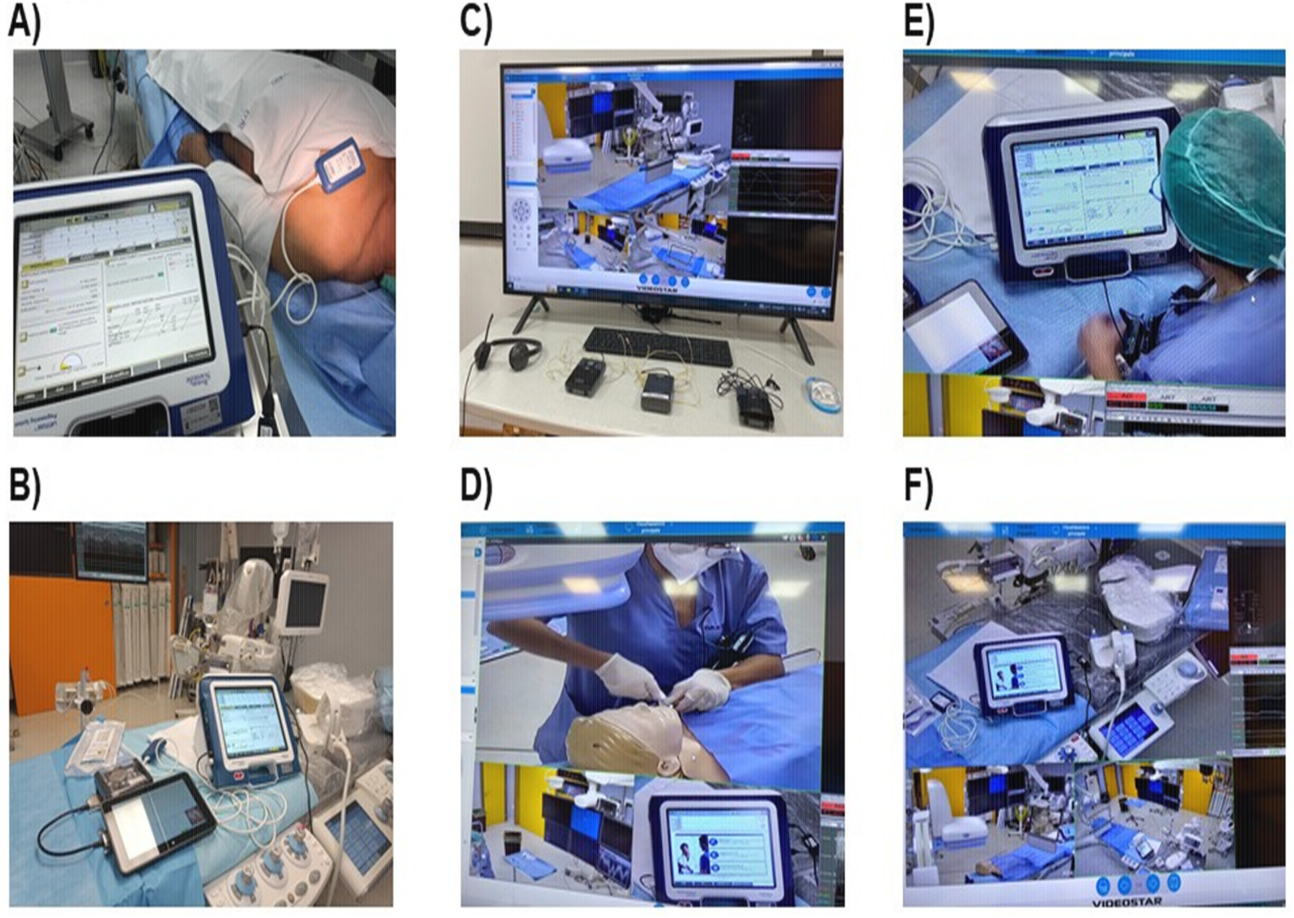
Remote FCS ysupport system setup. Representative pictures of remotely-driven procedures steps: **(A)** device interrogation and programming in a previously implanted patient; **(B)** external simulator displaying cardiac arrhythmias analyzed both in- and out-side the EP lab; **(C)** audio/video consolle set-up for remote guidance; **(D)** phantom covered by sterile drape; **(E)** physician operating in the EP lab while receiving instructions by the engineer at the computer; **(F)** rotating camera for focusing and zooming unlimited times in order to allow remotely-guided devices implantation.

A remote technical support system was developed to address various issues and to eliminate barriers toward specialized cardiac care for patients at two cardiology academic hospitals. A dedicated computer room with a multimedia information system was created to remotely guide ICD/CRT-D implantations (Figure 1). The technical setup for telemedicine-driven procedures included:

• A 16 inch video processor on the Videostar network (a video processing system manufactured by Videostar Security, based in Misterbianco, ITALY)
• Three Pan-Tilt-Zoom (PTZ) cameras with multiple rotating options and 8× magnification at the edges of the EP lab and the capability to move left and right (pan), up and down (tilt), allowing for comprehensive and adjustable coverage of the EP lab surface area.
• One centrally located PTZ camera with multiple rotating options, 360-degree yaw rotation, 30× magnification, and ceiling support video output on the POE network
• Three HDMI encoders over IP compatible with the video processor
• Three HDMI converters for various medical equipment video outputs
• An 8-port POE switch
• Videostar remote management vision software
• A QR1 Xilica main frame with 8 in/out cards, a DANTE 4 × 4 interface, two XCSML Xilica mic/line input cards, three XCSLO Xilica line output cards
• A GM9781H Bespeco UHF wireless microphone headset with 100 channels
• An amplified speaker with wall support
• An installation console running Windows 10, equipped with a Core i7-11th processor, Nvidia GeForce 8GB, 16GB DDR4, 1TB SSD, and additional cables, networks, and connectors for backup requirements.

### 2.1 First phase: assessment of telemedicine

Patients (*N* = 20) with previously implanted ICD/CRT-D devices, who were admitted for other invasive cardiology procedures, were asked to participate in a preliminary phase. The study involved testing audio-visual connections between the electrophysiologist in the EP lab and the FCS in the control station. This was done by visualizing the programmer screen (LATITUDE 3,300 programming system, Boston Scientific, St. Paul, MN, USA) and sharing decisions about device configuration and electrode parameters via web connection. Additionally, the ability to take clinical action against theoretical cardiac arrhythmias was tested using a dedicated simulator (Epicardio Ltd, Richmond, Surrey, UK). This phase aimed to test the technical functionality of remote assistance, ensure effective communication, and provide training in a controlled environment.

### 2.2 Second phase: telemedicine in interventional procedures

The final preclinical step was to perform ICD/CRT-D implantation into phantoms (*N* = 20) while receiving indications and suggestions from the FCS remotely connected. The phantom (Practi-Man Advance, SoFraPa, Calenzano, ITALY) was used for approaching all types of materials and equipment that were going to be used in the actual patient population. Since phantoms cannot be exposed to x-rays, we could not observe nor test the electrode implantation sites once placed into the body; instead, these tests and observations were reproduced using a dedicated simulator to replicate the necessary procedural steps and assess their functionality. This phase was conducted to ensure the practicality of remote support in real procedures, assessing its impact on device optimization, procedural efficiency, and safety. Phantoms were used to exclude possible concerns without exposing patients to potential risk in this initial phase.

### 2.3 Third phase: telemedicine-driven implantation procedures

Following baseline assessment, thirty consecutive patients (*N* = 30) eligible for ICD/CRT-D implantation were enrolled from June 2022 to September 2022 (Table 1) and randomized in standard procedure with engineer in the EP lab or procedure conducted with remote technical support.

**Table 1:** Clinical characteristics of all enrolled patients.

Clinical characteristics	All enrolled patients	Standard group procedures	Remote group procedures	*P* value
Age, years (mean ± SD)	64 ± 11	64 ± 11	65 ± 11	0.73
Male, *n* (%)	21 (70)	14 (70)	7 (70)	1.0
NIDCM, *n* (%)	12 (40)	8 (40)	4 (40)	1.0
IDCM, *n* (%)	15 (50)	10 (50)	5 (50)	1.0
Previous PCI, *n* (%)	13 (43)	8 (40)	5 (50)	0.71
CABG, *n* (%)	3 (10)	2 (10)	1 (10)	1.0
EF, % (mean ± SD)	35 ± 10	35 ± 11	33 ± 2	0.33
AF, *n* (%)	8 (27)	6 (30)	2 (20)	0.68
VA, *n* (%)	3 (10)	2 (10)	1 (10)	1.0

Values are mean ± SD, or *n* (%).

AF, atrial fibrillation; CABG, coronary artery bypass grafting; EF, ejection fraction; HCM, hypertrophic cardiomyopathy; IDCM, ischemic dilated cardiomyopathy; NIDCM, non-ischemic dilated cardiomyopathy; PCI, percutaneous coronary intervention; SD, standard deviation; VA, ventricular arrhythmia.

Inclusion criteria were patients undergoing ICD or CRT-D implantation with signed written informed consent, both genders and in range 18 ÷ 85 years of age.

Indications for ICD (Perciva™, Boston Scientific, St. Paul, MN, USA), CRT-D (Resonate, Boston Scientific, St. Paul, MN, USA) and subcutaneous-ICD (S-ICD, Emblem, Boston Scientific, St. Paul, MN, USA) were considered according to current guidelines for sudden cardiac death (SCD) (14) primary and secondary prevention (in case of ICD and S-ICD) (15, 16), or heart failure (HF) management (in case of CRT-D) on top of optimal medical therapy (17). PM-dependent patients were excluded due to longer and unpredictable procedural strategy (12), as well as generator replacements procedures that would not require fluoroscopy in majority of cases compared to *de novo* implantations.

Main endpoints were:

- Procedure success, defined as completion of the implantation with optimal lead placement and device activation.

Each procedure was considered complete when electronic parameters of all the implanted leads had been deemed optimal according to the clinical requirements: atrial and ventricular pacing thresholds <1,0 Volt at 0.5 ms pulse width, atrial sensing >1.0 mV, ventricular sensing >5.0 mV, and lead impedance between 300 and 1,500 ohms. For CRT-D devices, the left ventricular pacing threshold was defined as <2.5 Volts at 0.5 ms pulse width, with biventricular pacing synchronization adjusted for obtaining an optimal hemodynamic response, which is considered of paramount importance in HF (18). These goals were employed to confirm the feasibility of the remote procedure. Such assessments were performed by FCS during standard approach, or by non-scrubbed personnel when FCS in telemedicine was indicating how to use the dedicated programmer through the camera.

- Incidence of technical failure during follow-up, including lead dislocation, generator malfunction, or other technical issues requiring reintervention.

Other endpoints were evaluated in order to identify further advantages provided by telemedicine:

- Fluoroscopic time (FT) in telemedicine-assisted and standard procedures was assessed.
- Total occupancy time of the EP lab. For each procedure of procedural duration (in minutes) with telemedicine-assisted and standard approaches was performed.
- Stability of the electronic parameters was assessed to validate the reliability of the remote procedure. Telemetry checks of the implanted devices were performed by clinicians at bedside prior to discharge, and follow-up data were collected via RM at one, six months, and further ongoing.

All devices were provided by the same constructor, that ensured the widest portfolio of implantable devices at the time of this randomized trial (Boston Scientific, St. Paul, MN, USA).

All patients provided additional consent for monitor displaying of their clinical profiles and open discussion through ambient microphone and speakers of the most adequate devices to be implanted during the procedures.

This trial was conducted and reported according to Consolidated Standards of Reporting Trials (CONSORT) guidelines (19), hence approved by the Institutional Review Board.

## 3 Statistical analysis

Continuous data are expressed as mean ± standard deviation. Categorical variables were reported as counts and percentages, with comparisons made using the chi-square test or Fisher's exact test. The Shapiro–Wilk test was used to assess the normality of continuous variables and Levene's test for homogeneity of variance tests. Mann–Whitney *U* or Unpaired *t*-tests tests were assessed to compare data from radiologic exposure, procedural time and differences of outcome variables between standard approach and telemedicine-driven settlement as appropriate. The analyses were performed using the bootstrap technique with 5,000 resamples to estimate 95% confidence intervals. Bootstrap was employed to ensure robust estimates due to the small sample size. Statistical analysis was performed using IBM SPSS (v23, IBM Corp., Armonk, NY, USA). For all analysis, a *P* value < 0,05 was considered significant.

## 4 Results

### 4.1 Telemedicine-driven device implantation

Clinical characteristics of total enrolled population are described in Table 1. There were no statistically significant differences between the two groups. Before proceeding with real implantations in patients, all the procedural phases were handled by discussing electrocardiographic rhythm alterations, and electronic parameters of the leads and devices at distance by using dedicated simulator. Afterwards, the “patient preparation” phase which is commonly realized by covering with sterile drapes to create and maintain an adequate aseptic field during the procedure, was reproduced on a phantom, and usual procedural steps were finally executed.

### 4.2 ICD/CRT-D implantation through remote guidance

Ten clinical cases were performed by utilizing the monitoring platform that provides real-time support, and twenty patients were implanted while engineer was physically in the EP lab; more in detail, the telemedicine group consisted of one single-chamber ICD, two dual-chamber ICDs, four S-ICDs, three CRT-D devices, while the procedures list with onsite FCS accounted two single-chamber ICDs, three dual-chamber ICDs, twelve S-ICDs, three CRT-Ds. All procedures in both study arms were successfully completed (100% of cases).

### 4.3 Telemedicine reduces procedural duration of ICD/CRT-D implantation

**Figure 2:**
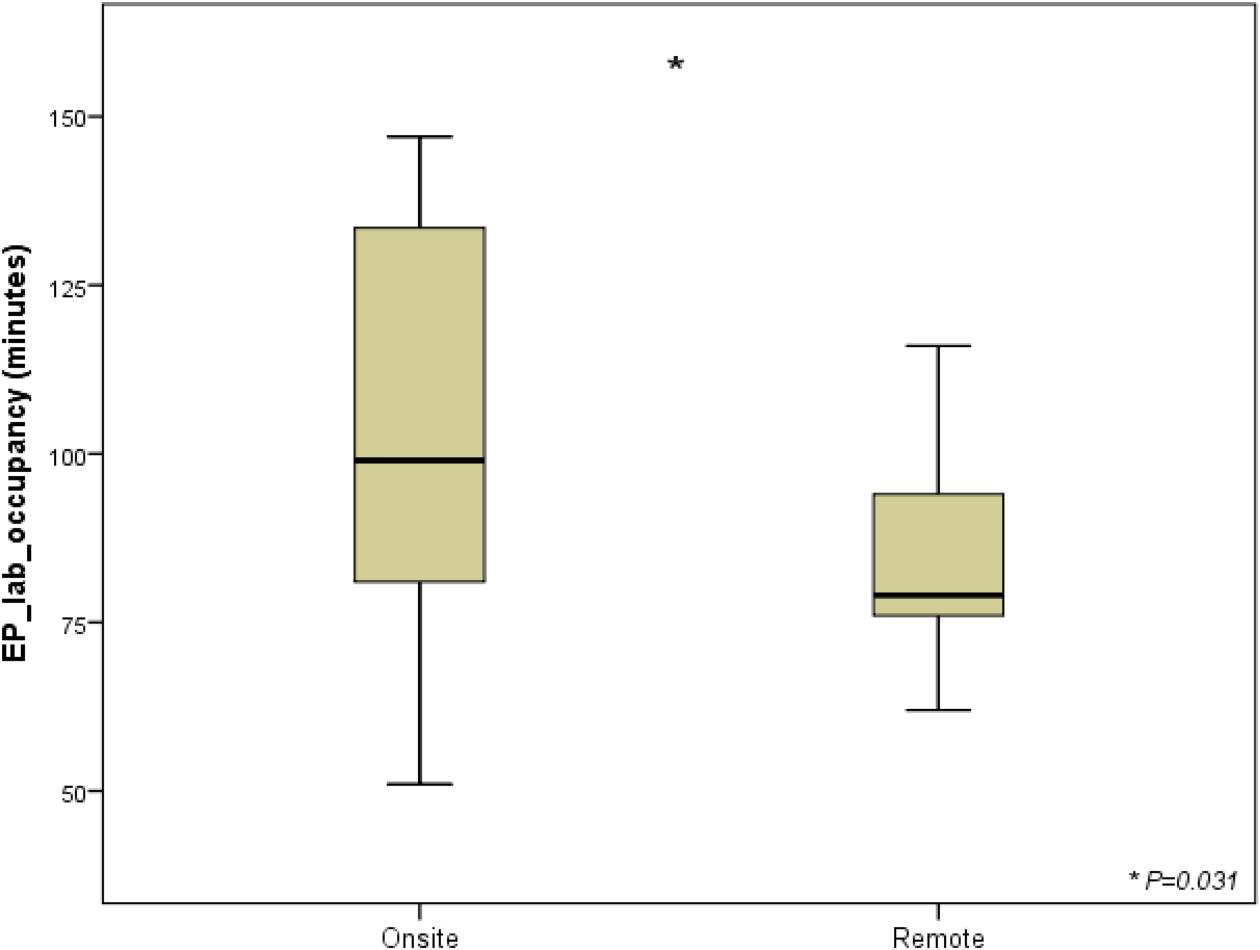
Total procedure length according to typology of cardiac implantable electronic devices. Box plots compare EP lab occupancy times (in minutes) between two groups: procedures with a biomedical engineer either physically on-site (*N* = 20) or providing remote telemedicine support (*N* = 10) during transvenous implantable cardioverter/defibrillator procedures (TV-ICD, 5 vs. 3), cardiac resynchronization therapy with defibrillators (CRT-D, 3 vs. 3), and subcutaneous ICD (S-ICD, 12 vs. 4). The EP lab occupancy time is longer for the onsite group, whereas the remote group shows shorter and more consistent occupancy times, suggesting greater efficiency with remote support. List of abbreviations: CRT-D, cardiac resynchronization therapy with defibrillator; EP lab, electrophysiology laboratory; ICD, implantable cardioverter defibrillator; S-ICD, subcutaneous; TV-ICD, transvenous implantable cardioverter defibrillator.

Each procedure required two separate connections, the first being related to discussing indications to implantation with materials to be used; at this time, physicians were not scrubbed. After aseptic washing and scrubbing, the second connection was related to supporting the actual procedure. Average durations of the EP lab occupancy with standard approach were 1 h 43 min ± 28 min and 1 h 24 min ± 6,5 min (*P* = 0,031) with telemedicine (Figure 2), considering an average duration of the connections via VIDEOSTAR™ platform of 60 min ± 5 min.

### 4.4 Comparison of radiation exposure between standard approach and telemedicine-driven implantation

**Figure 3:**
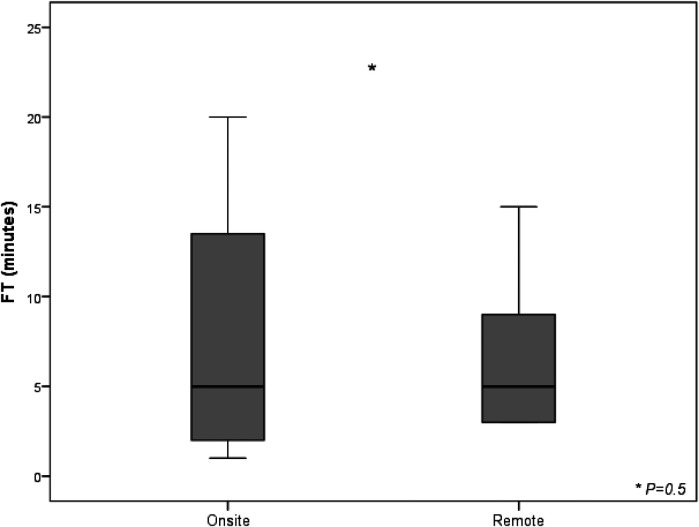
Fluoroscopy time and radiation exposure deriving from standard approach and from telemedicine-driven implantation. The box plots compare fluoroscopy times (in minutes) between two groups: cases with a biomedical engineer either physically on-site or providing remote telemedicine support. The interventions include transvenous implantable cardioverter-defibrillators (TV-ICD, 5 on-site vs. 3 remote), cardiac resynchronization therapy with defibrillators (CRT-D, 3 on-site vs. 3 remote), and subcutaneous ICDs (S-ICD, 12 on-site vs. 4 remote). The onsite group shows longer fluoroscopy times, while remote group has shorter and more consistent times. List of abbreviations: CRT-D, cardiac resynchronization therapy with defibrillator; ICD, implantable cardioverter defibrillator; S-ICD, subcutaneous implantable cardioverter defibrillator; TV-ICD, transvenous implantable cardioverter defibrillator.

Considering skin-to-skin time as superimposable to the duration of the connection between cardiac electrophysiologist in the EP lab and FCS in the control room, the further step to address the impact of this new setting was fluoroscopic time (FT) calculation. A reduction close to the threshold of statistical significance was observed in FT for procedures performed in telemedicine (6 min ± 4 min vs. 8 min ± 8 min, *P* = 0,5) considering the whole analyzed cohort undergone implantation with FCS either on site or under remote guidance (Figure 3).

### 4.5 Yield of telemedicine according to long-term performances

The physicians interviewed at the end of the procedures acknowledged the importance of the dedicated training and simulations before performing the actual interventions, even though critical issues and/or clinical emergencies were not encountered. Strikingly, RM of the implanted devices did not find technical failure in any part (electrode system nor generator) in any patient as indicated by sensing and pacing parameters assessed at one year of follow-up. Comparing leads electronic parameters in the multiple follow-up tests simultaneously between the two groups (telemedicine FCS and on-site FCS) no appreciable variations were revealed (Supplementary Table S1). Furthermore, as previously stated, all electronic parameters remained in the normal range in all cases (20, 21).

## 5 Discussion

We investigated the impact of remote guidance through FCS expert in implanting devices for cardiac rhythm control not joining the EP team during consecutive procedures of *de novo* implantations of ICD, S-ICD, and CRT-D.

Our current work is intended as implementation of digital health to cardiac rhythm management; we aimed at providing EP physicians an innovative service for implanting device by creating a support hub located proximal to Cath and EP labs. Moreover, the present study gained non-clinical impacts as well (22), since patient satisfaction was improved, and travel costs were saved as already demonstrated in previous experiences in which telemedicine was otherwise used only after discharge (23). Importantly, the above discussed approach was not related in any moment to any breach in cybersecurity since the setting proposed by our research group was solely based on direct screen visualization and continuous voice support.

We observed that time of occupation of the EP lab varies significantly between telemedicine-guided procedures and procedures with FCS on site; on parallel, x-ray requirement was slightly reduced in telemedicine group, probably because clinicians believed FCS was “constantly watching and driving” the actions of advancing, pushing, and torquing each implanted electrode. We might argue that the length of implant procedures was shortened by remote support because uninterrupted supervision of the procedures by the control station—as opposed to electrical cardioversion, ablations, and EP studies—associated with the physical FCS's on-site involvement.

Although there may not be an obvious process by which FT is decreased in telemedicine-driven cases, there are several potential contributing elements. Telemedicine offers ensuring reliable, on-demand technical expertise from virtually any location. Any technological problems or complications that surge during the intervention can be swiftly resolved. Moreover, fluoroscopy settings can be enhanced with a console that has a video camera with “multi-point” observation, resulting in more effective visualization and shorter fluoroscopy times. By having access to remote expert support, the on-site team may feel more confident in their ability to execute promptly the necessary adjustments, which could result in a shorter radiation use. Additionally, web-based real time support can improve overall efficiency by streamlining the workflow and reducing the delays. Given that CRT-D implants are considered demanding and time-consuming procedures worldwide, the above mentioned benefits are particularly evident in this study. Thus, our clinical experience demonstrates once again that to move toward a zero-fluoroscopy, advancements in technology are required as well as availability of skilled personnel.

This study demonstrates the feasibility of remote FCS guidance for ICD/CRT-D implantation. All procedures were successfully completed, and no major technical failures were observed at one-year follow-up. These findings provide the basis for larger-scale studies to evaluate long-term clinical outcomes and safety with higher statistical power.

The recent COVID-19 pandemics forced cardiology divisions worldwide (8, 24), and cardiac electrophysiology groups as well (25), to reduce their clinical activities (26), not just because of devoting resources to face the infectious disease, but also because qualified engineers allowed to programming the device for cardiac rhythm control were not allowed to enter in multiple hospitals in the same day, or were sick themselves, or just being told of a contact with an asymptomatic COVID-19 patient, that obliged them not to eventually contaminate the EP lab they were supposed to access upon call. At our center, FCS are typically present for these operations, collaborating with specialized nurses, technicians, and electrophysiologists to ensure the best procedure outcomes. Tests of the electronic parameters of the leads and the optimization of energy delivery by the device were the most requested consultations. Moreover, moving this setup to a more rural location or during a pandemic may render technical help available almost anywhere.

Interestingly, total occupation of the EP lab was significantly reduced, without any failure of the implanted electrodes, nor records of major adverse cardiovascular events when the FCS was guiding remotely the implantation procedure; on the other hand, we observed that defibrillation tests were not performed.

Previous work has shown that post discharge in-person visits can be replaced by virtual visits after hospitalization for HF (27). While this strategy was based primarily on patients compliance to appointments and drugs for managing HF, and other studies clearly showed the superiority of remote device monitoring for HF (28, 29), yet a gap in knowledge exists about the role of telemedicine in supporting device-based treatment for HF (1, 2); in this regard, our study addressed the feasibility of implanting ICD and CRT-D on top of optimal medical therapy (30), by implementing a telemedicine-based procedural stage.

This work underlines the need for safety in the EP lab that is provided by multiple skills, including FCS. This is translated into reduced x-rays for each procedure, and in need for additional qualified personnel when procedures in young patients are planned or in case defibrillation test is needed for secondary SCD prevention. Not all physicians are comfortable in performing such procedures without FCS on site; besides, an anesthesiologist is mandatory for assisted ventilation and vital parameters check during arrhythmia in case it is sustained, or even worsens.

While this study witnessed the transition from FCS to remote clinical specialist, further studies with biomedical engineer located only at distance need to be designed.

We recognize that connectivity is the current challenge in telemedicine worldwide (31), and that among requirements for sustained telehealth expansion a broadband internet access is of crucial importance (32). In a near future, taking into account the availability of remote interactive consoles, smart glasses, and software that bring augmented reality straight into the programmer, it is conceivable that the setting described in our report would be probably overcome. However, for those rural areas of the world yet uncovered by digital transmissions of data, high-speed internet connections and optic fibers, our system can provide workflow optimization through remote ICD/CRT-D implantation, followed by remote patient management (33, 34).

In the attempt to standardize the feasibility and safety of performing complex cardiovascular procedures without FCS into the EP lab, for security reasons the engineer was physically inside the hospital, adopting a connection established through ethernet cable (35). Future studies are warranted to address precision and accuracy of a reliable connection through satellite, which allows high-speed connectivity (36).

## 6 Limitations

This study has several limitations. The small sample size and short follow-up duration may not capture all potential differences or rare adverse events. The limited range of clinical scenarios excludes some patient groups, affecting the generalizability of the findings.

Additionally, the study was conducted in two primary care cardiac centers in Italy, limiting the generalizability to other regions.

Finally, programmed delivered shock was performed in few cases (3/30, 10%), which happened to be all in the S-ICD arm with FCS on site; at this point, we do not believe that such discrepancy between the studied groups could impact the message of the current work, but it reinforces the assumption that in severe clinical scenarios, such as secondary prevention of SCD (14), and in other HF cases with high burden of comorbidity a broader team including FCS is pivotal for obtaining a successful therapy.

## 7 Conclusions

Our findings suggest that subcutaneous and transvenous ICD, and CRT-D implantation is feasible in EP lab under a new perspective, in which dynamics are handled just by nurses, technicians, and clinicians without on-site FCS. The remote FCS support system proved functional and valuable for clinicians, providing specialist technical support in a timely manner. Further studies are required for safety implementation and eventual transfer of remote guidance of ICD/CRT-D surgery to rural community hospital settings or when facing pandemics.

## Data Availability

The raw data supporting the conclusions of this article will be made available by the authors, without undue reservation.
