# Supplementary material for: Real-time technical support for guiding remotely ICD/CRT-D implantation
Source: Front Cardiovasc Med. 2025 Mar 7;12:1525151. doi: 10.3389/fcvm.2025.1525151 (PMC11925861; doi:10.3389/fcvm.2025.1525151)
Supplement: Supplementary file 1 [file Table1.docx]

**Supplementary Table 1. Selective leads comparisons for lead implanted with FCS in telemedicine**

| **Electronic parameters RV lead** | **Discharge (mean±SD)** | **1-month FU (mean±SD)** | **6-months FU (mean±SD)** |  |
| --- | --- | --- | --- | --- |
| Sensing (mV) | 16.83±6.41 | 17.5±6.41 | 17.97±5.22 | P=0.95 |
| Threshold (V@0.4ms) | 0.42±0.12 | 0.42±0.098 | 0.55±0.08 | P=0.06 |
| Impedance (Ω) | 487.83±42.72 | 498.33±43.09 | 446±27.18 | P=0.07 |
| **Electronic parameters RA lead** | **Discharge (mean±SD)** | **1-month FU (mean±SD)** | **6-months FU (mean±SD)** |  |
| Sensing (mV) | 3.38±0.42 | 3.5±0.53 | 4.7±1.64 | P=0.12 |
| Threshold (V@0.4 ms) | 0.84±0.49 | 0.86±0.48 | 0.6±0.16 | P=0.54 |
| Impedance (Ω) | 523.2±135 | 518±73.28 | 549.6±92.8 | P=0.88 |
| **Electronic parameters LV lead** | **Discharge (mean±SD)** | **1-month FU (mean±SD)** | **6-months FU (mean±SD)** |  |
| Sensing (mV) | 19±5.57 | 17.47±8.4 | 18.13±7.97 | P=0.97 |
| Threshold (V@0.5 ms) | 2.33±0.7 | 2.2±0.72 | 1.37±0.38 | P=0.19 |
| Impedance (Ω) | 992.67±322.44 | 1300±458.26 | 1202.33±78.53 | P=0.54 |

**List of abbreviations:** FU= follow-up; LV= left ventricle; ms= millisecond; mV= millivolt; RA= right atrial; RV= right ventricle; SD = standard deviation; V= Volt; Ω= Ohm.
